# Metabolism and Metabolic Inhibition of Xanthotoxol in Human Liver Microsomes

**DOI:** 10.1155/2016/5416509

**Published:** 2016-03-10

**Authors:** Zhongnv Ma, Xianbao Shi, Gang Zhang, Feng Guo, Lina Shan, Jiqun Cai

**Affiliations:** ^1^Department of Pharmaceutical Toxicology, School of Pharmacy, China Medical University, Shenyang 110001, China; ^2^The First Affiliated Hospital of Liaoning Medical University, Jinzhou 121001, China; ^3^Department of Medicinal Chemistry, Virginia Commonwealth University, Richmond, VA 23219, USA

## Abstract

Cytochrome p450 (CYP450) enzymes are predominantly involved in Phase I metabolism of xenobiotics. In this study, the CYP450 isoforms involved in xanthotoxol metabolism were identified using recombinant CYP450s. In addition, the inhibitory effects of xanthotoxol on eight CYP450 isoforms and its pharmacokinetic parameters were determined using human liver microsomes. CYP1A2, one of CYP450s, played a key role in the metabolism of xanthotoxol compared to other CYP450s. Xanthotoxol showed stronger inhibition on CYP3A4 and CYP1A2 compared to other isoenzymes with the IC_50_ of 7.43 *μ*M for CYP3A4 and 27.82 *μ*M for CYP1A2. The values of inhibition kinetic parameters (Ki) were 21.15 *μ*M and 2.22 *μ*M for CYP1A2 and CYP3A4, respectively. The metabolism of xanthotoxol obeyed the typical monophasic Michaelis-Menten kinetics and *V*_max_, *K*_*m*_, and CL_int_ values were calculated as 0.55 nmol·min^−1^·mg^−1^, 8.46 *μ*M, and 0.06 mL·min^−1^·mg^−1^. In addition, the results of molecular docking showed that xanthotoxol was bound to CYP1A2 with hydrophobic and *π*-*π* bond and CYP3A4 with hydrogen and hydrophobic bond. We predicted the hepatic clearance (CL_*H*_) and the CL_*H*_ value was 15.91 mL·min^−1^·kg^−1^ body weight. These data were significant for the application of xanthotoxol and xanthotoxol-containing herbs.

## 1. Introduction

**Figure 1:**
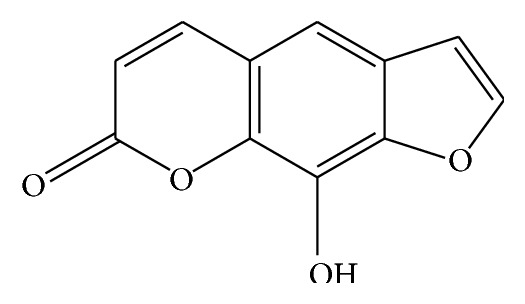
The structure of xanthotoxol.

Xanthotoxol (Figure 1), a biologically active linear furocoumarin, occurs in a large number of plants and is mainly extracted from the fruit of* Fructus Cnidii* [1]. Xanthotoxol shows strong pharmacological activities as anti-inflammatory, antioxidant, 5-HT antagonistic, and neuroprotective effects [2–4]. Respecting so many pharmacological activities of xanthotoxol, xanthotoxol is likely used with other drugs, and the possibility of herb-induced toxicity should be paid more attention. Among all of the metabolic processes, metabolisms catalyzed by the cytochrome P450 (CYP450) enzymes are the most important because almost 70%–80% of the known Phase I metabolisms are attributed to them [5]. Some of herbal medicines may result in CYP-mediated herb-drug interactions (HDIs) with prescribed other drugs [6]. For example, lovastatin was a substrate of cytochrome P450 3A4 (CYP3A4), and clarithromycin was an inhibitor of CYP3A4. When clarithromycin and lovastatin were coadministered, clarithromycin inhibited the activity of CYP3A4 and increased the serum concentrations of lovastatin and subsequent elevated the risk of myopathy [7]. In recent years, using humanized enzyme to study the drug-drug interactions in vitro has avoided the species differences of enzyme's isoforms, expression, and activities [8, 9]. The US FDA has already confirmed the validity of in vitro enzymes to assess the in vivo interaction between medications [10, 11].

In this study, we examined the inhibitory potential of xanthotoxol on CYP450s and kinetic parameters using in vitro human liver microsomes (HLMs), which will provide the basis for further in vivo studies in future.

## 2. Materials and Methods

### 2.1. Materials

Xanthotoxol (purity > 98%) was purchased from Sichuan Weikeqi Biotechnology Co. Ltd. (Sichuan, China). Paclitaxel, 1-aminobenzotriazole (ABT), phenacetin, sulfaphenazole, chlorzoxazone, quinidine, clomethiazole, furafylline, 8-methoxypsoralen, coumarin, diclofenac, quercetin, dextromethorphan, ketoconazole, testosterone, S-mephenytoin, omeprazole, glucose-6-phosphate dehydrogenase, NADP^+^, and D-glucose-6-phosphate were obtained from Sigma-Aldrich (St. Louis, MO, USA). All other reagents were the highest purity commercially available or HPLC grade.

### 2.2. Preparation and Characterization of Liver Microsomes

Liver microsomes from human (HLMs) used in this study were provided by the Research Institute for Liver Disease Co. (Shanghai, China). The HLMs were prepared from eleven individual human donor livers. Protein concentration and microsomes activities of CYP2C19, CYP2A6, CYP2C8, CYP2D6, CYP1A2, CYP2C9, CYP2E1, and CYP3A4 had been previously characterized by the Research Institute for Liver Disease Co.

### 2.3. CYP450 Probe Substrate Assays

HLMs phenacetin o-deethylation, coumarin 7-hydroxylation, paclitaxel 6*α*-hydroxylation, diclofenac 4′-hydroxylation, dextromethorphan o-demethylation, chlorzoxazone 6-hydroxylation, testosterone 6*β*-hydroxylation, and S-mephenytoin 4′-hydroxylation activities were utilized as selective markers for CYP1A2, CYP2A6, CYP2C8, CYP2C9, CYP2D6, CYP2E1, CYP3A4, and CYP2C19.

### 2.4. Incubation System for Liver Microsomes or Recombinant CYP450 Supersomes

The optimal conditions for microsomal incubation were described previously [12]. The incubation mixture, with a total volume of 200 *μ*L, consisted of 100 mM potassium phosphate buffer (pH 7.4), an NADPH-generating system (10 mM glucose 6-phosphate, 1 unit/mL of glucose 6-phosphate dehydrogenase, and 4 mM MgCl_2_), liver microsomes (0.3 mg/mL), or recombinant CYP450s (15 nM). In all experiments, xanthotoxol was serially diluted to the required concentrations and the final methanol concentration did not exceed 1% (v/v) in the mixture. After a 5 min preincubation at 37°C, the NADP^+^ (1 mM, 20 *μ*L) was added into the mixture to initiate the reaction. The reaction was terminated by the addition of methanol (200 *μ*L) with internal standard. The mixture was kept on ice until it was centrifuged at 20 000 ×g for 10 min at 4°C. Aliquots of supernatants were stored at −40°C until analysis. Control incubations without NADPH or without substrate or without microsomes were carried out to ensure that the formation of metabolites was microsomes and NADPH dependent. All incubations throughout the study were carried out in at least three independent experiments with standard deviations (SD) generally below 10%.

### 2.5. CYP450s Inhibition Experiments

Inhibition study was conducted using the above incubation system consisting of liver microsomes, NADPH-generating system, probe substrate at the concentration of about *K*_*m*_ value, and xanthotoxol (or the control inhibitor). The concentrations of positive inhibitors used were as follows: 10 *μ*M furafylline for CYP1A2, 2.5 *μ*M 8-methoxypsoralen for CYP2A6, 10 *μ*M quercetin for CYP2C8, 10 *μ*M sulfaphenazole for CYP2C9, 10 *μ*M quinidine for CYP2D6, 50 *μ*M clomethiazole for CYP2E1, 20 *μ*M omeprazole for CYP2C19, 1 *μ*M ketoconazole for CYP3A4, and 500 *μ*M ABT for broad CYP450s. Kinetic analysis was performed where activity has been inhibited by more than 90%. Half inhibition concentration (IC_50_) value was obtained by incubating various xanthotoxol concentrations (0–100 *μ*M for CYP1A2 and CYP3A4). The *K*_*i*_ value was obtained by incubating various xanthotoxol and probe substrates concentration.

### 2.6. Kinetic Study

To estimate kinetic parameters of xanthotoxol and make sure that the formations of metabolites were in the linear range of both reaction time and the concentration of microsomes, xanthotoxol (0.25–200 *μ*M) was incubated with pooled HLMs for 30 min. Reaction velocities and substrate concentrations were used to calculate the apparent *K*_*m*_ and *V*_max_ values according to nonlinear regression from the Michaelis-Menten equation (see (1)). CL_int_ was calculated as *V*_max_/*K*_*m*_. In addition, the result was graphically represented on Eadie-Hofstee plots (velocities versus ratios of velocities to substrate concentrations) to determine whether the metabolism was monophasic or biphasic. Consider

(1) $$\begin{array}{c} V=\frac{V_{max}\cdot \left[S\right]}{K_{m}+\left[S\right]}, \end{array}$$

where *V*_max_ was the maximum reaction velocity, *K*_*m*_ was the Michaelis constant that represented the substrate concentration at which the velocity was half of *V*_max_, *V* was the reaction velocity, and [*S*] was the substrate concentration.

### 2.7. Prediction of In Vivo Hepatic Clearance

The following equations were used to predict the xanthotoxol clearance in human [13]:

(2) CLint  in  vitro=VmaxKm,CLint  in  vivo=CLint  in  vitro·SF,CLH=QH·fu·CLint  in  vivoQH+fu·CLint  in  vivo,

where SF (scaling factor) represents the milligrams of microsomal protein per gram of liver multiplied by the grams of liver weight; CL_int_ is the intrinsic metabolic clearance; CL_*H*_ is hepatic clearance; *f*_*u*_ is the free fraction in blood (there are no data of xanthotoxol; here *f*_*u*_ was arbitrarily proposed to be 1); *Q*_*H*_ is the hepatic blood flow. The hepatic clearance of xanthotoxol was calculated using (2), and physiological parameters in human were described as follows: microsomal protein per gram of liver, liver weight per kilogram of body, and liver blood flow for human were 48.8 mg, 25.7 g, and 20.7 mL·min^−1^·kg^−1^, respectively [14].

### 2.8. Molecular Docking Analysis

The X-ray crystal structures of human CYP1A2 (pdb: 2HI4) and CYP3A4 (pdb: 4K9W) were obtained from RCSB Protein Databank (http://rcsb.org/). The ligands for docking were prepared using SYBYL X2.1, and the energy was minimized using the external Tripos force field. The protonation state and energy minimization of the protein and the ligands were calculated using the default setting in SYBYL X2.1. The active sites were defined by a sphere of 6.0 Å from the native ligands in the crystal structures using Gold v5.2. The docked poses were scored using CHEMPLP scoring function. The best docked pose of the ligand was visualized using Pymol Molecular Graphics System v1.3.

### 2.9. HPLC Method

The HPLC system (Shimadzu, Kyoto, Japan) consisted of a CBM-20Alite system controller, two LC-20AB pumps, and an SPD-20A ultraviolet light (UV) detector. The chromatographic separation was achieved using a C18 column (4.6 mm: 150 mm, 5 mm Kromasil). The mobile phases consisted of LC grade water containing 0.1% formic acid (A) and LC grade acetonitrile (B) with the following gradient profile: 0–12 min, 20% B; 12-13 min, 20–95% B; 13–19 min, 95% B; 19-20 min, 90–20% B; 20–25 min, 20% B. The flow rate was 1 mL/min. Detection wavelength was set at 310 nm and the column temperature was set to 40°C.

## 3. Results

### 3.1. Analysis of Xanthotoxol Metabolites

**Figure 2:**
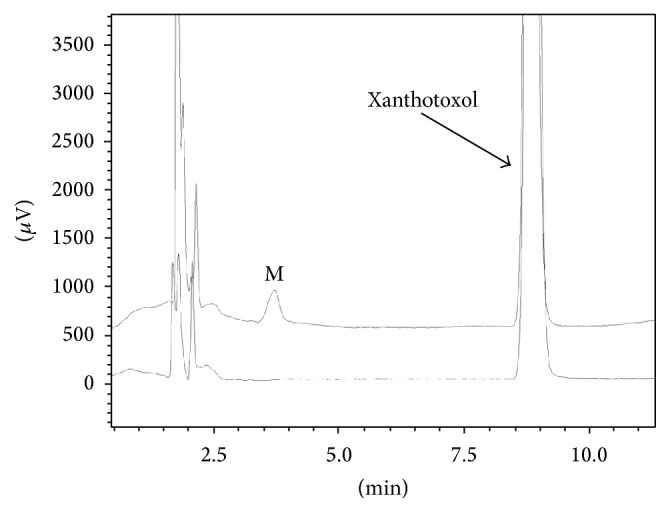
Chromatogram of incubation of xanthotoxol with human liver microsomes (HLMs). Xanthotoxol (10 *μ*M) was incubated with HLMs (0.3 mg/mL) at 37°C for 60 min with (upper) or without (lower) a *β*-nicotinamide adenine dinucleotide phosphate- (NADPH-) generating system.

After the incubations of xanthotoxol (10 *μ*M) in HLMs and NADPH-generating system, the new peak (M) was found at 3.7 min (Figure 2). The new peak was not observed in the negative controls without NADPH, or without substrate, or without microsomes.

### 3.2. Identifying CYP450s Involved in the Metabolism of Xanthotoxol

**Figure 3:**
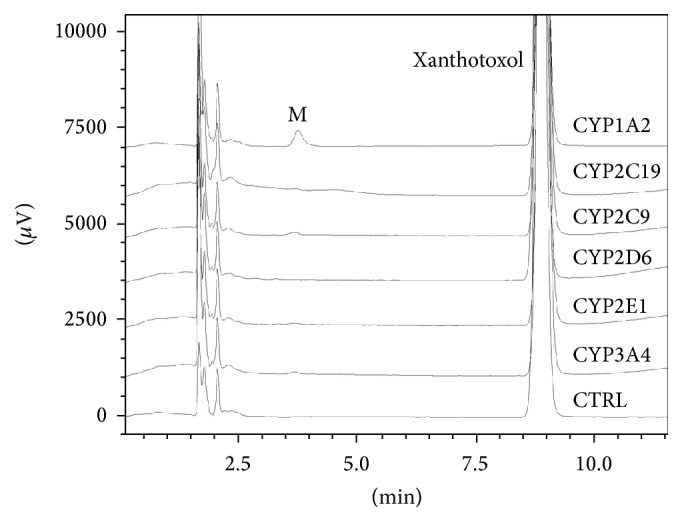
Representative HPLC profiles of xanthotoxol and its metabolite in recombinant CYP450 supersomes. Xanthotoxol (10 mM) was incubated with recombinant CYP450 supersomes (15 nM) and an NADPH-generating system at 37°C for 30 min.

The enzymes involved in the xanthotoxol metabolism were investigated using cDNA-expressed human P450 isoforms including CYP3A4, CYP2D6, CYP2E1, CYP2C9, CYP2C19, and CYP1A2. The incubation for each isoenzyme was carried out as described for the liver microsomal study. As shown in Figure 3, the metabolite (M) was mainly generated in the presence of CYP1A2, and the amounts of M generated by CYP3A4, CYP2D6, CYP2E1, CYP2C9, and CYP2C19 were 4.5%, 0%, 7.3%, 13.5%, and 3.6% comparing to CYP1A2 level which was normalized to 100%.

### 3.3. Inhibition of Xanthotoxol against CYP3A4 and CYP1A2

**Figure 4:**
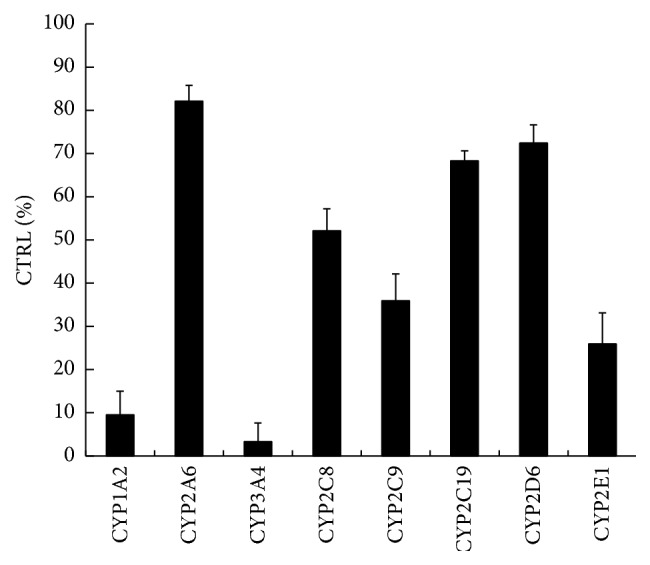
The inhibitory effects of xanthotoxol on CYP450s in human liver microsomes (HLMs).

**Figure 5:**
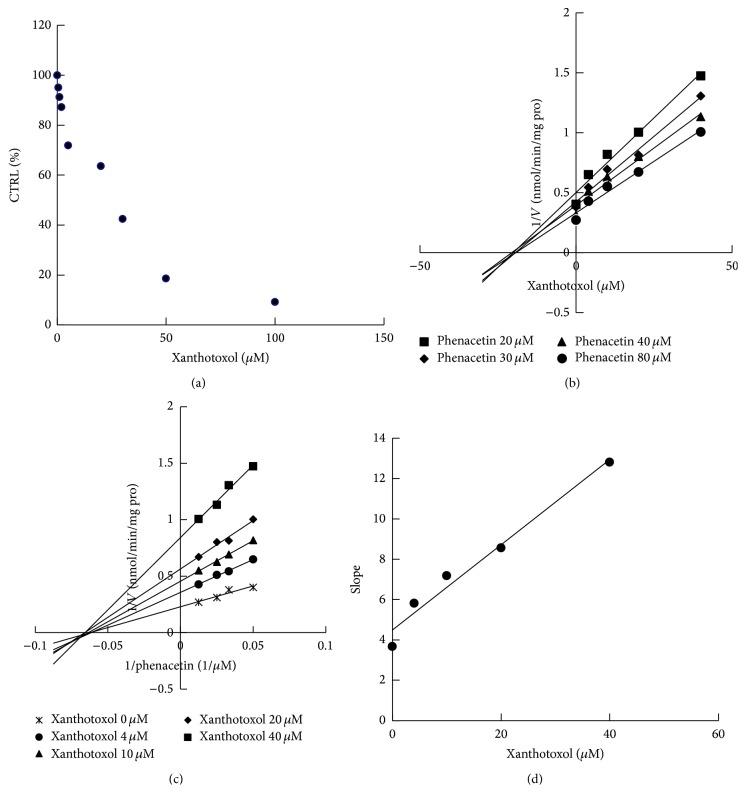
(a) Inhibitory effects of xanthotoxol on phenacetin o-deethylation activity (CYP1A2). (b) Dixon plot of inhibition effect of xanthotoxol on phenacetin o-deethylation (CYP1A2). (c) Lineweaver-Burk plot of inhibitory effect of xanthotoxol on phenacetin o-deethylation (CYP1A2). (d) Secondary plot of slopes from Lineweaver-Burk plot versus xanthotoxol concentrations. Each data point represented mean of triplicate incubations.

**Figure 6:**
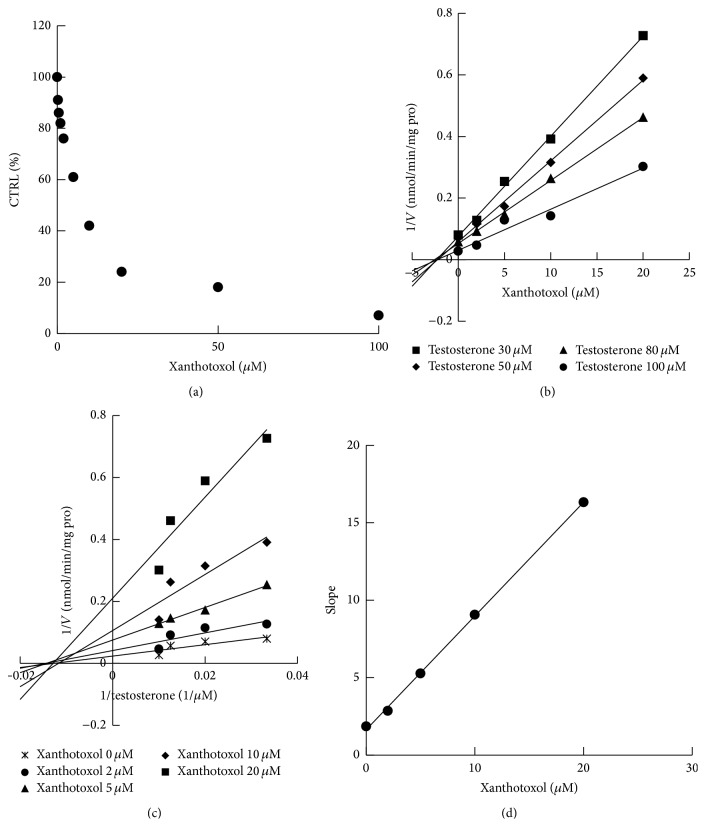
(a) Inhibitory effects of xanthotoxol on testosterone 6*β*-hydroxylation activity (CYP3A4). (b) Dixon plot of inhibition effect of xanthotoxol on testosterone 6*β*-hydroxylation (CYP3A4). (c) Lineweaver-Burk plot of inhibitory effect of xanthotoxol on testosterone 6*β*-hydroxylation (CYP3A4). (d) Secondary plot of slopes from Lineweaver-Burk plot versus xanthotoxol concentrations. Each data point represented mean of triplicate incubations.

All positive control inhibitors performed strong inhibition to the corresponding probe reactions with more than 80% of control activity inhibited. 100 *μ*M xanthotoxol inhibited the activities of CYP3A4, CYP2C9, CYP1A2, CYP2A6, CYP2D6, CYP2C8, CYP2C19, and CYP2E1 by 3.3, 35.9, 9.5, 82.1, 72.4, 52.1, 68.3, and 25.9%, respectively (Figure 4). Furthermore, kinetic analysis of CYP3A4 and CYP1A2 was performed. As shown in Figures 5 and 6, xanthotoxol inhibited diclofenac 4′-hydroxylation (CYP1A2) and testosterone 6*β*-hydroxylation (CYP3A4) in a concentration-dependent manner with the IC_50_ of 27.82 *μ*M for CYP1A2 and 7.43 *μ*M for CYP3A4. Lineweaver-Burk and Dixon plots showed that the inhibition of xanthotoxol to CYP1A2 and CYP3A4 was best fit to a noncompetitive way. The *K*_*i*_ value was calculated to be 21.15 and 2.22 *μ*M for CYP1A2 and CYP3A4, respectively.

### 3.4. Kinetic Characteristics of Xanthotoxol Metabolism

**Figure 7:**
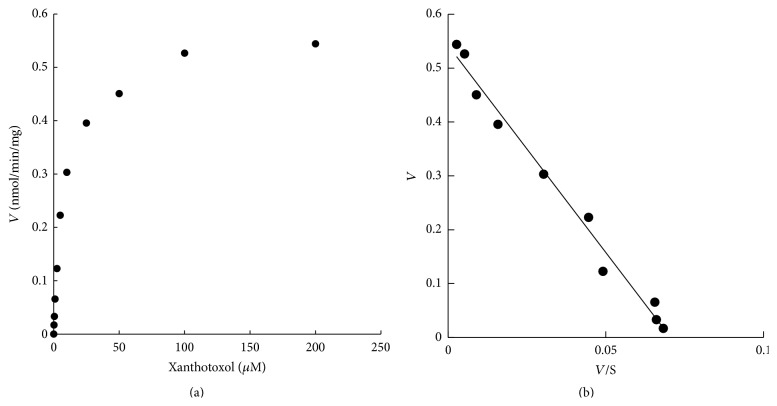
Michael-Menten plots (a) and Eadie-Hofstee plots (b) of xanthotoxol metabolism in human liver microsomes (HLMs). Incubation conditions were carried out as described in Section 2. Each point represents the mean of three independent experiments in triplicate determinations.

Over the whole concentration range tested, the metabolism of xanthotoxol obeyed the typical monophasic Michaelis-Menten kinetics, as evidenced by the Eadie-Hofstee plot (Figure 7). Reaction velocity showed significant concentration-dependent characteristic. *V*_max_ and *K*_*m*_ values were calculated as 0.55 nmol·min^−1^·mg^−1^ and 8.46 *μ*M via nonlinear regression from the Michaelis-Menten equation using Origin software. Intrinsic clearance (CL_int_) was 0.06 mL·min^−1^·mg^−1^.

### 3.5. Prediction of In Vivo Hepatic Clearance of Humans

Using the values of kinetic parameters generated in HLMs, CL_*H*_ was calculated to be 15.91 mL·min^−1^·kg^−1^ body weight. The percentage of CL_*H*_ versus hepatic blood flow (*Q*_*H*_)% was 76.8%.

### 3.6. Molecular Docking Study

**Figure 8:**
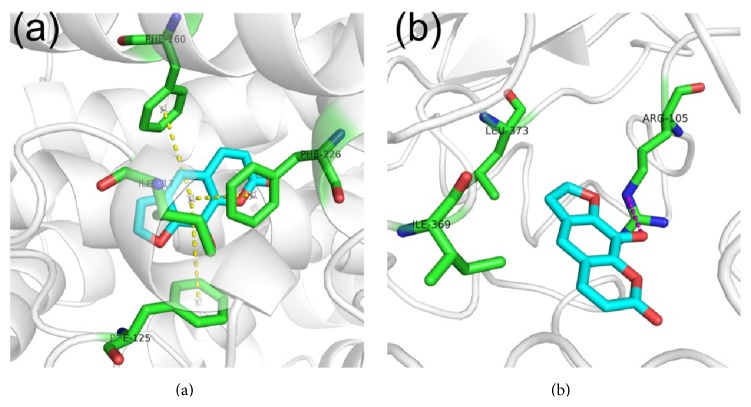
Binding modes of compound with CYP 1A2 (a) and 3A4 (b). The compound was displayed in cyan stick, residues were displayed in green sticks, hydrogen bond was displayed in purple dotted line, and *π*-*π* stacking was displayed in yellow dotted line.

As shown in Figure 8(a), there was hydrophobic effect between xanthotoxol and CYP1A2 with ILE117. In addition, the benzene rings of xanthotoxol also showed *π*-*π* stacking interaction with PHE125, PHE226, and PHE260. For docking to CYP3A4 (Figure 8(b)), xanthotoxol was bound to CYP3A4 via hydrogen bond with ARG105 and hydrophobic interactions with ILE369 and LEU373.

## 4. Discussion

Cytochrome P450 enzymes (CYP450s) are heme-thiolate proteins that are responsible for the oxidative metabolism of numerous xenobiotics as well as endogenous substrates [15]. The inhibition against CYP450s has resulted in costly late failures of drug development and withdrawal of drugs on the market [16]. Thus, estimating HDIs is a priority to predict inhibitory potential of major components on CYP450 isoforms and to clarify the inhibition mechanism and kinetics.

Using pooled HLMs, we determined the inhibitory effects of xanthotoxol on eight CYP450 isoforms and the results showed that xanthotoxol exhibited a stronger and noncompetitive inhibition to CYP1A2-mediated phenacetin o-deethylation and CYP3A4-mediated testosterone 6*β*-hydroxylation with the IC_50_ values of 27.82 *μ*M and 7.43 *μ*M, respectively. The inhibition kinetic parameters (*K*_*i*_) were calculated to be 21.15 *μ*M and 2.22 *μ*M for CYP1A2 and CYP3A4, respectively. The metabolite (M) of xanthotoxol was detected in HLMs and obeyed the typical monophasic Michaelis-Menten kinetics. The generation of the metabolite was NADPH dependent. Aminobenzotriazole (ABT), a nonspecific inhibitor of cytochrome P450s (CYPs), showed potent inhibition against metabolite production, which proves that metabolism was catalyzed by CYP450s. The kinetic characteristics of xanthotoxol metabolism were 0.55 nmol·min^−1^·mg^−1^ and 8.46 *μ*M for *V*_max_ and *K*_*m*_. CL_int_ was 0.065 mL·min^−1^·mg^−1^. According to the Eadie-Hofstee plots, xanthotoxol metabolism showed a monophasic feature, suggesting that xanthotoxol was metabolized by only one isozyme or two isozymes with the same *K*_*m*_ value. CYP3A4 is the most important CYP isoform for human and metabolizes approximately 50% of marketed drugs [17]. Thus, potential metabolism-based herb-drug interactions might occur during coadministration of xanthotoxol with other CYP3A4 or CYP1A2 substrates.

In this study, we identified the CYP450 isoforms involved in xanthotoxol metabolism by screening assays with recombinant CYP450 supersomes including CYP1A2, CYP3A4, CYP2E1, CYP2C9, CYP2D6, and CYP2C19. CYP1A2 contributed main effect to catalyze the formation of metabolite of xanthotoxol, while other enzymes displayed a very limited ability to metabolize xanthotoxol. Thus, xanthotoxol was selectively metabolized by CYP1A2.

Hepatic clearance is considered to be one of the most important pharmacokinetic parameters as it directly relates to drug elimination and bioavailability. So a prediction of hepatic metabolic clearance is of primary importance during the drug discovery and development process. In this study, we investigated the quantitative prediction of human hepatic metabolic clearance from in vitro experiments using human liver microsomes focusing on CYP450s metabolism. CL_*H*_ was calculated to be 15.91 mL·min^−1^·kg^−1^ body weight. In general, drugs that have CL_*H*_ above 70%  *Q*_*H*_ are classified as high-clearance drugs and those below 30%  *Q*_*H*_ are classified as low-clearance drugs [18]. So we predicted from in vitro data that xanthotoxol was high-clearance drug in human body.

Analyzing molecular docking, we found that xanthotoxol was bound to CYP1A2 with hydrophobic interaction and *π*-*π* stacking and CYP3A4 with hydrogen bonds and hydrophobic interaction, which implied that xanthotoxol exhibited stronger interaction with CYP3A4 than CYP1A2. The enzyme kinetic studies confirmed the molecular docking study that xanthotoxol had lower IC_50_ and *K*_*i*_ values against CYP3A4 than CYP1A2.

It is important to understand the pharmacokinetic profiles of xanthotoxol and its drug interaction with CYP450s. Our results indicate that xanthotoxol inhibited the activity of CYP1A2 and CYP3A4 and therefore may interact with drugs that were metabolized by these two isozymes.
